# Eco-Friendly Lithium Separators: A Frontier Exploration of Cellulose-Based Materials

**DOI:** 10.3390/ijms25136822

**Published:** 2024-06-21

**Authors:** Tian Zhao, Pengcheng Xiao, Mingliang Luo, Saiqun Nie, Fuzhi Li, Yuejun Liu

**Affiliations:** School of Packaging and Materials Engineering, Hunan University of Technology, Zhuzhou 412007, China; xpc111294@163.com (P.X.); westbrook123666@163.com (M.L.); 15874987968@163.com (S.N.); culaisett@126.com (F.L.)

**Keywords:** cellulose, battery separator, lithium-ion battery, preparation process, modification method

## Abstract

Lithium-ion batteries, as an excellent energy storage solution, require continuous innovation in component design to enhance safety and performance. In this review, we delve into the field of eco-friendly lithium-ion battery separators, focusing on the potential of cellulose-based materials as sustainable alternatives to traditional polyolefin separators. Our analysis shows that cellulose materials, with their inherent degradability and renewability, can provide exceptional thermal stability, electrolyte absorption capability, and economic feasibility. We systematically classify and analyze the latest advancements in cellulose-based battery separators, highlighting the critical role of their superior hydrophilicity and mechanical strength in improving ion transport efficiency and reducing internal short circuits. The novelty of this review lies in the comprehensive evaluation of synthesis methods and cost-effectiveness of cellulose-based separators, addressing significant knowledge gaps in the existing literature. We explore production processes and their scalability in detail, and propose innovative modification strategies such as chemical functionalization and nanocomposite integration to significantly enhance separator performance metrics. Our forward-looking discussion predicts the development trajectory of cellulose-based separators, identifying key areas for future research to overcome current challenges and accelerate the commercialization of these green technologies. Looking ahead, cellulose-based separators not only have the potential to meet but also to exceed the benchmarks set by traditional materials, providing compelling solutions for the next generation of lithium-ion batteries.

## 1. Introduction

As one of the most popular energy storage technologies today, easily rechargeable lithium-ion batteries currently dominate the portable electronics market [1]. Besides electronics, lithium-ion batteries are gaining importance in the military, electric vehicles, and aerospace [2]. Lithium-ion batteries have many advantages, such as high working voltage, high specific energy density, low self-discharge, long cycle life, no memory effect, fast charging and discharging, and no environmental pollution. They can be used not only for laptops and mobile phones but also for smart cars, large-scale power supplies, and flexible wearable electronic devices [3,4]. As an important component of lithium batteries, separators play an important role in battery safety. Thus, improving the performance of battery separators is an important approach to enhancing battery safety and electrochemical performance [5]. In recent years, there has been a steady increase in the number of scientific reports on battery separators. This indicates that the application of battery separators is very promising. However, the practical application value of battery separators also needs to consider the feasibility of large-scale manufacturing and the associated costs. Although many reviews have summarized different categories of cellulose-based lithium-ion battery separators [6,7], they often overlook the discussion and analysis of the synthesis methods [8] and manufacturing costs [9] of these separators. Based on this, we conducted an in-depth survey of the polymer industry chain in Zhuzhou City, Hunan Province, China, and systematically analyzed and summarized the synthesis methods and manufacturing costs of various types of cellulose-based battery separators. This review focuses on the current production methods of cellulose-based battery separators, as well as the modification and development status of new battery separators. In addition, we also conducted an in-depth discussion on the future development direction of battery separators, aiming to provide valuable references and guidance for researchers and industry professionals in this field.

**Figure 1:**
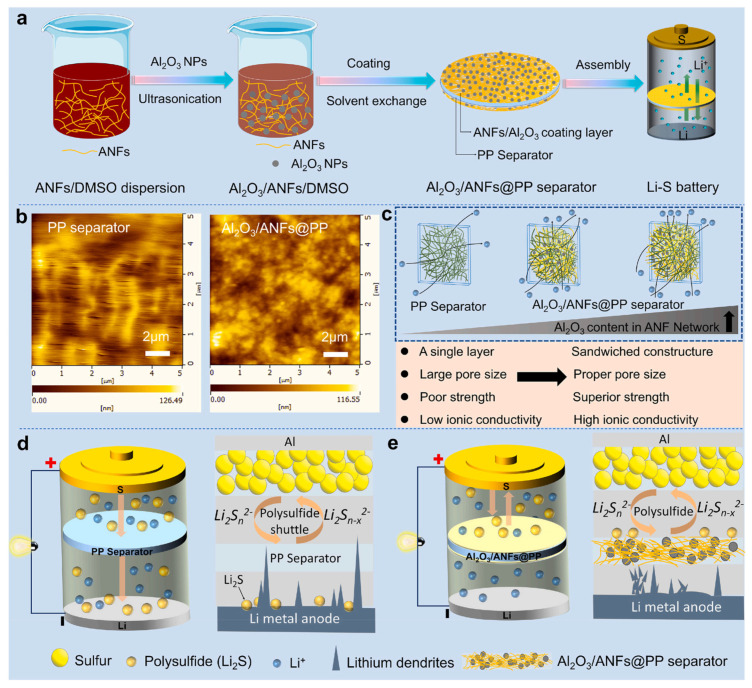
Schematic illustration of the composite separator based on ANF and Al_2_O_3_ [33]. (**a**) Preparation process. (**b**) AFM phase topographies of the separators. (**c**) Schematic diagram of the effect of Al_2_O_3_ NPs content on ionic transport of the separator. Comparison of the mechanisms for blocking polysulfide shuttle and lithium dendrites between the PP (**d**) and Al_2_O_3_/ANFs@PP (**e**) separators. Copyright © 2022, with permission from Elsevier.

A battery separator refers to the polymer film between the positive and negative electrodes of a lithium-ion battery [10,11,12,13,14]. The battery separator isolates the positive and negative electrodes while preventing the battery from short circuits caused by direct contact between positive and negative electrodes. Its performance can directly affect the capacity, internal resistance, and interface contact area of the battery [15]. Polyethylene (PE) and polypropylene (PP) are currently the most widely used battery separators [16,17], with excellent chemical stability, low manufacturing costs, and no toxicity, and they are the preferred materials for lithium-ion battery separators on the market [18,19,20,21,22]. However, commercial polyolefin separators have disadvantages such as relatively low porosity, poor wettability, and poor thermal stability, which affects the cycling performance of the battery [23,24,25,26,27]. For this reason, researchers have conducted a lot of research on the modification of polyolefin separators, which can effectively improve the wettability of polyolefin separators to electrolyte solutions by grafting hydrophilic monomers on their surfaces or applying hydrophilic coatings [28,29,30,31,32]. For instance, Li and his coworkers [33] developed a sandwich-type separator based on aramid nanofibers (ANFs) and Al_2_O_3_ nanoparticles by a simple coating process on top of a polyolefin separator (Figure 1). The sandwich separator exhibited high strength, toughness, desirable nanoscale porosity, excellent thermal stability, and wettability. Zuo et al. [34] coated poly (vinylidene fluoride)/ethyl cellulose and amino-functionalized SiO_2_ nanocomposites on polyethylene separators; the contact angle of the separators decreased from 49.5° to 23.2°, and the ionic conductivity increased from 0.34 mS cm^−1^ to 0.79 mS cm^−1^. Although the electrolyte wettability and ionic conductivity of the separator were improved, the polyolefin materials would undergo severe thermal contraction under high-temperature conditions, leading to spontaneous combustion due to internal short circuits of lithium-ion batteries. Therefore, these types of separators are not suitable for use under high-temperature conditions.

Due to the insufficient heat resistance and weak wettability of commercial lithium-ion battery separators in electrolyte solutions, researchers have started to apply various natural and synthetic materials to lithium-ion battery separators. Natural materials mainly include cellulose and its derivatives, and synthetic materials mainly include polyethylene terephthalate (PET), polyvinylidene fluoride (PVDF), polyvinylidene fluoride-hexafluoropropylene (PVDF-HFP), polyimide (PI), aramid, etc. [35,36,37,38,39,40,41,42].

Cellulose materials are environmentally friendly polymers with low cost, abundant resources, and excellent wettability with electrolytes [43,44,45]. However, natural cellulose-based battery separators suffer from large pore size, low mechanical strength, and flammability under high-temperature conditions. Moreover, the crude cellulose fibers result in poor film-forming performance [46]. Therefore, natural cellulose needs to be modified to meet the requirements for lithium battery separators. In recent years, researchers have developed various modification strategies to improve the properties of cellulose-based battery separators, such as chemical modification, physical blending, surface coating, and nanocomposite formation.

A lot of research has been conducted on cellulose-based lithium battery separators in the academic field, and some review articles have discussed the application of some specific cellulose-based lithium battery separators. However, they have not systematically classified and discussed the relevant cellulose materials suitable for lithium batteries, especially in terms of the preparation methods of cellulose-based lithium battery separators, and the summary and analysis are not comprehensive enough. For example, Jabbour et al. [47] introduced the application of some specific cellulose materials in lithium battery cathode and anode materials as well as separators, but did not summarize and generalize the relevant battery performance of cellulose-based lithium battery separators. Hu [7] and colleagues’ review of lithium-ion battery separators focused mainly on synthetic materials, and their discussion was mainly based on polyimide (PI), with little involvement of cellulose and its derivatives, and no detailed evaluation of cellulose-based lithium battery separators. Lizundia and coworkers [6] focused on the discussion of lithium battery separators that contain cellulose acetate (CA), cellulose nanofibers (CNFs), and cellulose nanocrystals (CNCs), while other cellulose-based lithium battery separators were rarely mentioned. Xia and colleagues [8] conducted an in-depth analysis of the impact of functional groups on the performance of cellulose-based battery separators and clearly explained how functional groups enhance the mechanical strength, thermal stability, and electrochemical performance of the separators. However, although their work provides valuable insights into the role of functional groups in improving the performance of separators, the paper did not discuss the comparison between different functional group modification strategies in terms of synthesis methods in detail, nor did it address the specific impact of these modification methods on manufacturing costs. Similarly, Luo et al. [9] primarily focused on the structure, characteristics, preparation, modification, and performance of the separator, without delving into the discussion of manufacturing costs. In order to accelerate the commercialization process of cellulose-based battery separators, future research needs to consider these factors more comprehensively to optimize the synthesis process and reduce production costs. Therefore, this article provides a comprehensive review and summary of the recent research progress in cellulose-based lithium-ion battery separators, and systematically compares and analyzes the advantages and disadvantages of different preparation technologies, as well as the performance of the separators produced by these technologies, with a particular focus on cost-effectiveness. These in-depth analyses and insights undoubtedly provide valuable references and guidance for scholars who will conduct scientific research in this field in the future, contributing to the advancement and application of cellulose-based separator technology.

## 2. Lithium-Ion Battery Separators Based on Cellulose and Its Derivatives

Cellulose and its derivatives have attracted extensive research interest because of their unique properties, such as controllable porosity, excellent mechanical and thermal stability, non-toxicity, and inherent hydrophilicity; they are widely used in supercapacitors, batteries, sensors, and conductive materials [48,49]. The cellulose used in the lithium-ion battery separator can be classified as physically modified cellulose and chemically modified cellulose [50]. The physically modified cellulose including microcrystal cellulose (MCC) [51,52], micro-fibrillated cellulose (MFC) [53,54], and nanocellulose [55,56,57]. The chemically modified cellulose containing cellulose acetate (CA) [58,59,60], carboxymethyl cellulose (CMC) [61,62,63], methyl cellulose (MC) [64,65], ethyl cellulose (EC) [66,67], and hydroxy cellulose (HC) [68,69]. Table 1 shows the comparison of cellulose-based battery separator performance with PP and PE separators.

Compared to commercial polypropylene (PP) and polyethylene (PE) separators, cellulose-based lithium-ion battery separators offer several advantages. Firstly, cellulose is derived from natural materials, making it renewable and biodegradable, thus meeting environmental requirements. Secondly, its high thermal stability prevents shrinkage or melting at high temperatures, reducing the risk of thermal runaway in batteries. The hydrophilicity of cellulose allows for better electrolyte wettability, improving ion conductivity. Additionally, cellulose separators typically have high mechanical strength and toughness, effectively preventing short circuits and enhancing battery safety and longevity. Lastly, cellulose separators exhibit good compatibility with electrolytes and electrode materials, minimizing side reactions, reducing internal resistance, and extending battery life. These advantages make cellulose separators significantly promising for improving battery performance and safety. Table 2 shows the performance and cost comparison of polyolefin battery separators and cellulose-based lithium battery separators. The cost of various separators in the table refers to the price per 100 pieces and is all from the China market survey.

### 2.1. Physically Modified Cellulose

Physically modified cellulose is a special form of cellulose that is modified by physical methods to improve the physical properties of natural cellulose. Typically, the addition of physically modified cellulose to a lithium battery separator can improve the electrolyte wettability of the separator, which enhances the ionic conductivity and electrolyte absorption rate, and its battery cycling performance is also significantly increased [98]. However, compared with the traditional polyolefin battery separator, the mechanical properties of the physically modified cellulose-based separator will be reduced, the lithium dendrite inhibition capacity is relatively weak, and its preparation cost will be higher [99].

#### 2.1.1. Microcrystal Cellulose

**Figure 2:**
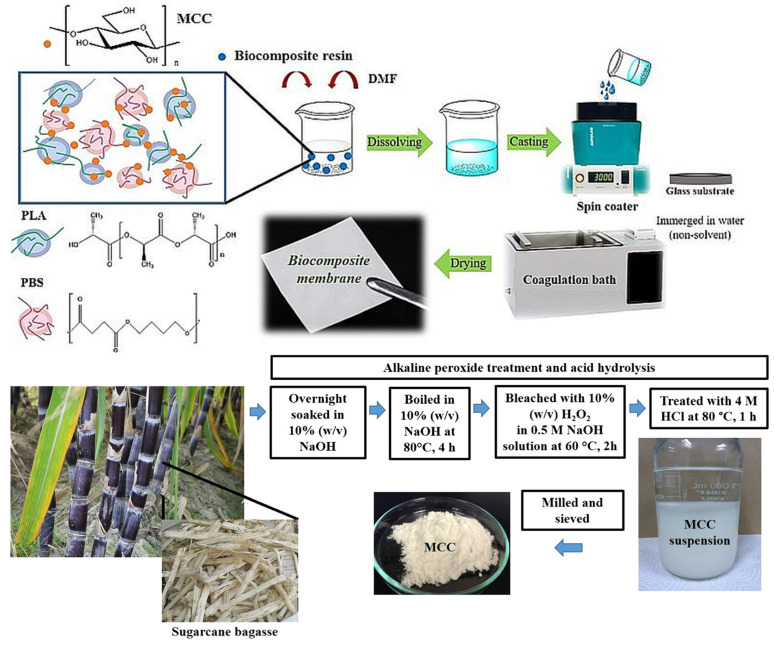
Specific process of MCC battery separator production [72]. Copyright © 2019, with permission from Springer Nature B.V.

Compared with natural fibers, microcrystal cellulose has better chemical inertia, higher thermal stability, and hygroscopicity; hence, it is widely used in the commercial field [100,101,102,103,104,105,106,107]. Modeling has revealed that microcrystalline cellulose has a good adsorption effect on anions and cations in the free state, which is mainly due to the electron-poor and electron-rich groups on microcrystalline cellulose [101]. Generally, MCC can be obtained from biomass materials through hydrolysis [108], with important characteristics such as biodegradability, high mechanical strength, large surface area, and low density [109,110,111]. Thus, it is a promising material to replace traditional polyolefin lithium battery separators. Thiangtham et al. [72] prepared an MCC-modified PLA/PBS composite battery separator using dimethyl formamide as a solvent and deionized water as a coagulation bath using the phase inversion method. The results showed that the thermal stability of the separator was the best when 5% solid MCC content was added. After heat treatment at 135 °C for 1 h, only 32% of the separator contracted, and the liquid absorption rate of the separator reached 138%, further improving the ion conductivity (Figure 2). Kanbua and colleagues [112] successfully prepared sulfonated cellulose (SC) from MCC extracted from sugarcane bagasse by gamma radiation and sulfonation with potassium metabisulfite. Then, they introduced SC into the (PEBAX/PEGDA) matrix as a reinforcing material and hydrophilic filler to improve the electrolyte affinity and thermal stability of their composite membranes. The increase of SC in the PEBAX/PEGDA composite membrane resulted in enhanced hydrophilicity, electrolyte uptake, and thermal stability compared to the original composite membrane.

#### 2.1.2. Micro-Fibrillated Cellulose

**Figure 3:**
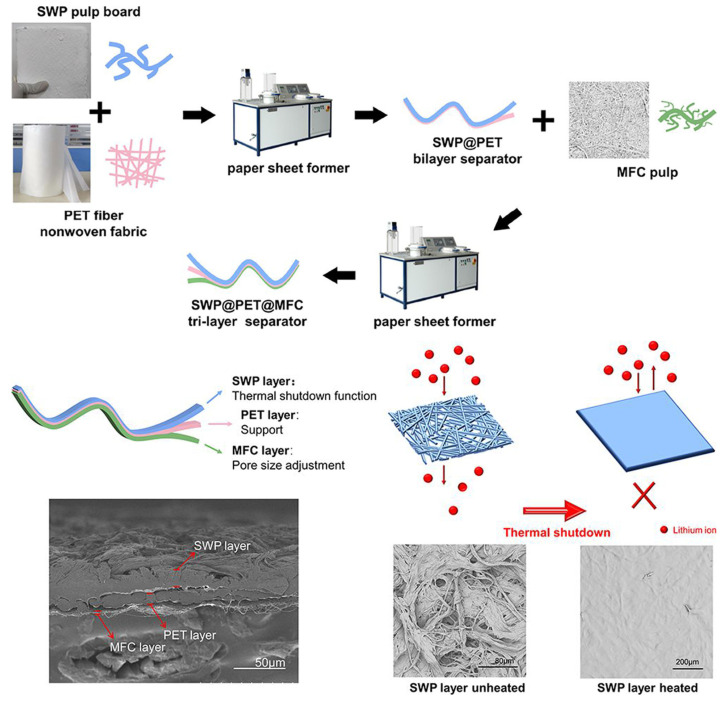
Schematic illustration for preparation of SWP@PET@MFC separators [71]. Copyright © 2023, with permission from American Chemical Society.

Micro-fibrillated cellulose (MFC) is usually obtained from natural cellulose through stirring and ultrasonic homogenization, and is a highly wettable colloidal substance with low preparation cost, excellent mechanical properties, and electrochemical stability, and its unique three-dimensional mesh structure can be used as a reinforcing body, which can effectively improve the mechanical strength of cellulose battery separators [113,114,115,116,117,118,119,120]. Micro-fibrillated cellulose (MFC) is a nanoscale cellulose material with a high aspect ratio, high surface area, and high hydroxyl content. MFC can be prepared by three main methods: chemical mechanical treatment, mechanical methods, and chemical acid hydrolysis. Among them, high-pressure homogenization is a widely used mechanical method that can produce MFC with good dispersion in water and compatibility with other materials [121]. MFC can be used as a reinforcing agent for methacrylic acid-based polymer electrolytes, which can enhance the mechanical, thermal, and electrochemical properties of battery separators. Moreover, MFC can be combined with various modified materials, such as graphene oxide, polyvinylidene fluoride, and polyethylene oxide, to form composite battery separators with improved performance. For example, Xu et al. [122] used dopamine as a modified material to blend the MFC suspension after pulping with dopamine solution, and then prepared a cellulose/polydopamine (CPD) battery separator using a papermaking mechanism by washing the slurry with water. The surface of the cellulose battery separator modified with dopamine is rich in dense nanopores, effectively improving the liquid absorption rate and mechanical strength. Similarly, to increase the battery separator porosity and improve the problems such as uneven thickness, Pan and coworkers [70] obtained a mesoporous Cladophora cellulose (CC) battery separator with good thermal stability using a simple manufacturing method. The experimental results indicate that the CC battery separator has good stability at 150 °C and exhibits electrochemical inertness in the range of 0–5 V. LiFePO_4_/Li batteries containing a CC separator exhibit good cycle stability and 99.5% discharge capacity retention after 50 cycles at a rate of 0.2 C. Zhao et al. [123] successfully prepared a new cellulose separator for lithium-ion batteries (LIBs) by TEMPO oxidation and vacuum filtration. The separator showed good tensile strength (75 MPa), excellent thermal dimensional stability, excellent porosity (64.8%), outstanding liquid electrolyte uptake (323%), and large ionic conductivity (2.83 mS cm^−1^). However, in the actual operation process, MFC will exhibit irreversible aggregation, which is caused by the increased proximity of cellulose chains during the drying process and the resulting intermolecular bonding. This process is called horrification, which usually leads to higher crystallinity, and thus stronger fibers. Due to the aggregation of micro/nano fibrils, the reinforcement potential of MFC is limited. To address this phenomenon of irreversible aggregation, Hiltunen and coworkers attempted to use a high-speed grinder for grinding and dispersing in the final step of dispersion, which improved the dispersion and uniformity of the MFC suspension [124]. Li et al. [71] prepared a three-layer structured non-woven separator (SWP@PET@MFC) with a thermal shutdown function by the papermaking method. The incorporation of micro-fibrillated cellulose fibers regulated the pore size of the composite separator and improved the wettability of the composite separator. The composite separator exhibits excellent thermal stability and a wide thermal shutdown temperature window, which greatly improves the safety of the battery (Figure 3).

#### 2.1.3. Nanocellulose

Nanocellulose has the advantages of a large specific surface area, high aspect ratio, and excellent mechanical properties [125,126]. The lithium battery separator made of nanocellulose features low cost, high porosity of the separator, excellent ion conduction performance, and high electrochemical stability [127,128,129,130,131]. Nanocellulose is usually classified as cellulose nanofibers (CNFs) [132,133,134], cellulose nanocrystals (CNCs) [135,136], and bacterial cellulose (BC) [137,138,139].

**Figure 4:**
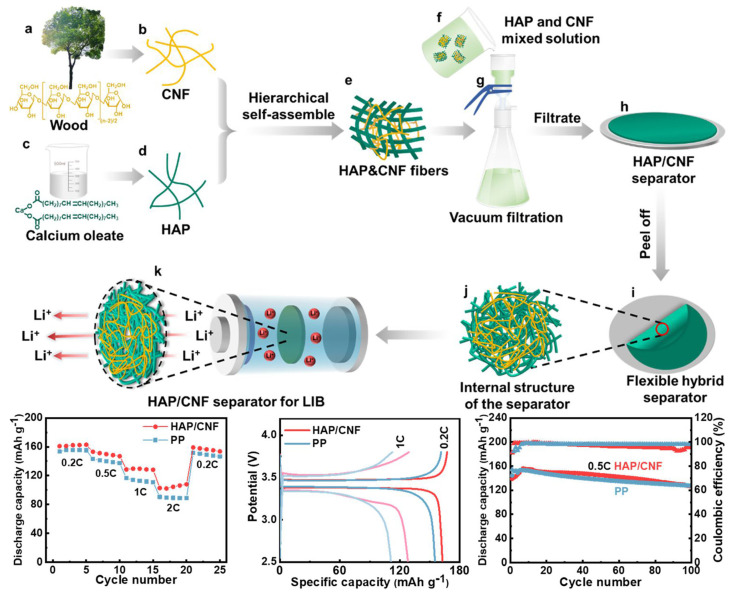
Fabrication of hydroxyapatite/cellulose nanofiber (HAP/CNF) hybrid separators and electrochemical performance [75]. (**a**) Starting raw materials (natural wood) for CNFs. (**b**) Fabricated cellulose nanofibers. (**c**) Precursor (calcium oleate) for hydroxyapatite nanowires. (**d**) Hydroxyapatite nanowires. (**e**) HAP and CNF network. (**f**) HAP and CNF mixed solution. (**g**) Vacuum filtration. (**h**) HAP/CNF hybrid separator. (**i**) Flexible hybrid separator peeled off from a filter membrane. (**j**) Internal structure of the separator constructed by HAP and CNFs. (**k**) The application of the HAP/CNF separators in LIBs. The hybrid separators are thermally stable and electrolyte-wettable, and the processes are green. Copyright © 2023, with permission from American Chemical Society.

CNFs are intertwined with each other to form a porous structure that facilitates ion transport. The surface of the fibers is also rich in hydrophilic groups such as hydroxyl and carboxyl groups, making them have good moisturizing ability for electrolyte solutions. Compared to traditional cellulose materials, CNFs have a high specific surface area, which can effectively improve their selective permeability to ions and their chemical activity. Moreover, CNFs have high strength and flexibility, which can enhance the mechanical properties and the bending resistance of the battery separator when used as a component. CNFs are a kind of natural source material, and therefore have better biodegradability, avoiding environmental pollution and harm. For instance, the nanocellulose/PET non-woven battery separator prepared by Zhang and coworkers [73] has an electrolyte absorption rate of 250% and a porosity of 70%. Nanocellulose used in lithium-ion batteries is renewable and has excellent thermal stability. In the drying process of nanocellulose separators, due to capillary action, the separators will shrink to form a dense structure, which affects the electrolyte retention capacity of the separators and prevents ion migration. To address this issue, Guo et al. [99] used the micron-scale pores generated by the dissolution of PS spheres to the highly porous nature of the battery separator. Although the resulting pores weakened the mechanical strength of the separator, the electrochemical properties including ionic conductivity, interfacial resistance, and electrochemical window were improved due to the unique microstructure. The battery performance (including capacity retention and multiplication capability) of the button cell assembled with the prepared battery diaphragm was determined to be satisfactory. Tert-butanol (TBA) has a small polarity, and its use as a dispersion medium for cellulose can effectively avoid the tight bonding between fibers due to hydrogen bonding. Wang and his colleagues [74] prepared a pure CNF separator with excellent performance by vacuum filtration using cellulose nanofibers (CNFs) as the matrix and tertiary butyl alcohol as the dispersing medium. In addition, the batteries assembled from the separator showed excellent cycling stability. Hydroxyapatite (HAP) has excellent strength and thermal stability with a melting point of 1650 °C. Ultra-long hydroxyapatite nanowires (HAP NWs) can easily form porous membranes with a three-dimensional network structure, which can be used as a high-temperature-resistant LIB separator, but their mechanical strength needs to be further improved. Liu et al. [75] prepared HAP/CNF separators by the vacuum filtration method after fully mixing an aqueous solution containing HAP nanowire network and CNF suspension. Compared with the PP separator, the electrolyte uptake, ionic conductivity, and mobility number of the HAP/CNF separator were enhanced. Furthermore, the raw materials of hydroxyapatite and cellulose are green, and the preparation process is more environmentally friendly (Figure 4).

Cellulose nanocrystals (CNCs) are rigid rod-like particles, which are shorter than cellulose nanofibers (CNFs). Cellulose nanocrystals (CNCs) are biocompatible, biodegradable, and renewable nanomaterials with high specific surface area, high mechanical strength, and abundant surface hydroxyl groups. These properties enable CNCs to act as accelerators for the transport of free-state ions in the battery electrolyte and to serve as reinforcing agents in lithium battery separators. Moreover, CNCs can self-assemble into various multiphase or higher-order structures when dispersed in polar solvents, which can be used as templates for the design of novel functional nanomaterials [140]. For instance, a PVDF/HFP/CNC nanocomposite battery separator was fabricated by Kelley et al. [141]. It was found that the incorporation of CNCs improves the tensile strength of the separator. Mittal et al. [40] grafted CNCs on the nanopores of a polyvinyl alcohol film after lithiation of CNCs. The Li battery diaphragm exhibited an electrolyte uptake of 510 wt% and ionic conductivity of 3.077 mS cm^−1^. Due to the use of organic electrolytes, the diaphragm was able to achieve stable lithium metal deposition without dendrite growth, providing 94 mAh g^−1^ in a 100 mAh g^−1^ Li/LiFePO_4_ cell after 200 cycles. Ajkidkarn and his colleagues [77] prepared BCNCs/PEBAX microporous membranes by nonsolvent induce phase separation by extracting bacterial cellulose nanocrystals from coconut waste and compounding them with polyether block amide (PEBAX). Sulfuric acid hydrolysis of BCNCs produces rod/needle-shaped BCNCs and negative surface charges, which are favorable for transporting lithium ions, and PEBAX has excellent flexibility, low surface resistivity, and chemical resistance, which are favorable for resisting lithium dendrite puncture. By embedding BCNCs, PEBAX can be provided with appropriate thermal and dimensional stability and enhanced electrochemical performance.

Bacterial cellulose (BC) is a nanoscale biopolymer with a porous mesh synthesized by microbial fermentation and is named bacterial cellulose because it is synthesized by bacteria. Bacterial cellulose (BC) is a biocompatible nanomaterial with high tensile strength that can replace synthetic fibers derived from fossil fuels. Its high surface area-to-volume ratio enables strong interactions with other components of BC-based composites. Its reactive hydroxyl and other groups allow for various chemical modifications to produce functionalized BC with a wide range of “smart” applications. The simplicity of the BC production process offers the potential for large-scale, low-cost applications. Furthermore, an emerging high-value application for BC is in electrochemical energy storage devices as battery separators [142]. BC exhibits high water-holding capacity and unique mechanical properties because of the 3D structure of the nanofibers [143,144]. Yang and colleagues [145] used traditional papermaking methods to uniformly mix BC with different contents of ANFs, with ANF contents of 2%, 4%, 6%, and 8%, respectively, to prepare ANF/BC battery separators, which exhibit high tensile strength and ionic conductivity. Huang et al. [38] used TEMPO-oxidized bacterial cellulose to prepare a TOBC nanofiber lithium battery separator. The TOBC nanofiber separator exhibited a superior electrochemical stability window (>6.0 V), high electrolyte uptake (339%), outstanding ionic conductivity (13.45 mS cm^−1^), and low interfacial resistance (96 Ω). In order to improve the drawbacks of bacterial cellulose separators, Zhang et al. [79] introduced an aqueous solution of water-soluble APP (ammonium phosphate) and LPC (a mixture of lignosulfonate and epichlorohydrin resins) into an aqueous dispersion of bacterial cellulose (BC), which was then freeze-dried and pressed to obtain a cellulose composite separator (BLA). The water-soluble APP improved the flame-retardant and tensile properties of the separator, and the good compatibility between BC, LPC, and APP improved the mechanical properties, porosity, and electrolyte absorption of the composite diaphragm. Batteries assembled from BC separator have excellent cycle stability.

### 2.2. Chemically Modified Cellulose

**Figure 5:**
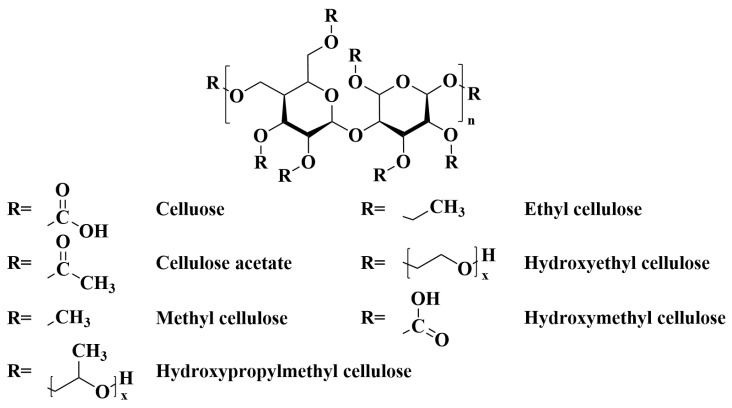
Chemical structure diagram of chemically modified cellulose.

Chemically modified cellulose involves the chemical treatment of natural cellulose to form special forms of cellulose, which have different compositions, structures, and properties and are widely used in pulp, electrochemistry, petrochemicals, food, pharmaceuticals, and other fields. The following are common chemically modified celluloses: cellulose acetate (CA), methyl cellulose (MC), carboxymethyl cellulose (CMC), ethyl cellulose (EC), hydroxy cellulose (HC), etc., and their chemical structures are shown in Figure 5. The chemical modification of cellulose involves introducing functional groups into the ordinary cellulose structure, which endows the cellulose with the relevant properties of the groups. When applied in lithium battery separators, this can greatly enhance the performance of lithium batteries. As shown in Table 2, chemically modified cellulose separators have advantages over traditional polyolefin separators in terms of electrolyte uptake, capacity retention, and ionic conductivity (up to a 3000% increase). The main drawback is the high preparation cost. Therefore, the future goal is to reduce the cost while maintaining good battery performance.

#### 2.2.1. Cellulose Acetate

Cellulose acetate (CA) is the most abundant and widely used renewable polymer resource due to its outstanding thermal stability, chemical resistance, and biodegradability [146]. Cellulose acetate (CA) exhibits higher hydrophilicity than other cellulose materials due to the presence of acetyl groups. Cellulose acetate-based battery separators can contact the electrolyte sufficiently, showing excellent wettability and improving the electrolyte uptake of the separator.

**Figure 6:**
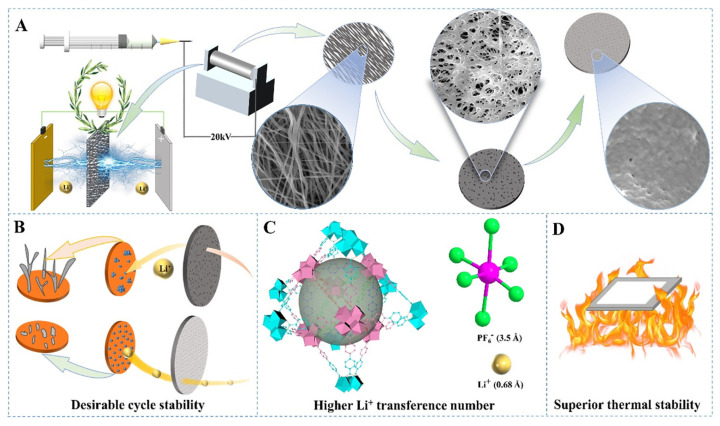
Schematic diagram of a layered network derived from 3D cellulose acetate with controllable nanopores [149]. Copyright © 2021, with permission from Elsevier.

Weng and coworkers prepared alkaline-treated cellulose acetate battery separators through electrospinning technology [147]. Under the microscope, acetate fibers exhibit a three-dimensional fiber network structure with random orientation, complete interconnection, and high porosity, with a porosity of up to 76%. Compared with commercially available polypropylene battery separators, cellulose acetate battery separators exhibit excellent thermal stability, electrolyte wettability, higher ionic conductivity, and better electrochemical stability. Hu et al. [148] prepared a battery separator by modifying a mixture of cellulose acetate (M-CA) and polyethylene glycol dimethyl acrylate (M-SiO_2_) through free radical polymerization. The mechanical strength of the separator was as high as 14.30 MPa, and there was no significant change in size at 200 °C. Meanwhile, the battery separator exhibited good ion conductivity, reaching 1.54 mS·cm^−1^. The LiFePO_4_/GPE/Li half-cell assembled from this membrane exhibited excellent cyclic charge–discharge performance and discharge performance, with a discharge-specific capacity retention rate of 98% and a discharge-specific capacity of over 100 mAh·g^−1^ after 100 cycles at 0.5 C. Deng and coworkers [149] designed and successfully prepared cellulose acetate-based lithium battery separators by a simple one-step process where the cellulose acetate backbone inhibited anion transfer and prevented large-scale accumulation of lithium ions, thereby limiting dendrite nucleation and growth. The 3D network separator exhibited 78.6% capacity retention after 900 cycles at 1C, with increases in elongation at break and strength of 620% and 28.4%, which resulted from the controlled porosity of nanofiber bridging (Figure 6). Cellulose acetate is too soluble in liquid electrolytes, and reducing the solubility of cellulose acetate can greatly improve battery performance. Liu et al. [81] prepared insoluble CPT separators by compounding CA, methylacryloyl chloride, poly (ethylene glycol) methyl ether methacrylate (PEGMEMA) and trihydroxymethylpropyl trimethylacrylaye (TMPTMA) via UV-initiated polymerization reaction. The polymer matrices are cross-linked with each other to improve the thermal and chemical stability of the separator, and the high content of electrolytic affinity groups and the dense cross-linked structure not only ensure the good ionic conductivity of the separator but also effectively improve the mechanical strength. Porous cellulose acetate separators were prepared using a combination of non-solvent and thermally induced phase separation (N-TIPS) by Arundati et al. [82]. The separator with a porous structure could be efficiently prepared by mass transfer between NMP as solvent and water as non-solvent. After the minimum evaporation time treatment, the separator had high ionic conductivity, porosity, and electrolyte uptake. Preparing cellulose acetate diaphragms with simultaneously high thermal stability and homogeneous porous structure improves their electrochemical performance and extreme environmental tolerance. Kung and his colleagues [150] used glycerol and sodium chloride as additives and prepared a nanoscale porous composite cellulose acetate separator with excellent thermal stability by adjusting their molar ratios. The results showed that thermal stability could be improved by stabilizing the coordination bond between the carbonyl group of CA and the remaining additives.

#### 2.2.2. Methyl Cellulose

Methyl cellulose (MC), a water-soluble cellulose derivative, is widely used as a commercial viscosity modifier and exhibits good mechanical properties and thermal stability when it is used in lithium-ion battery separators [151]. Methyl cellulose is formed by introducing methyl groups into cellulose. As a battery separator, methyl cellulose can increase the thermal and chemical resistance of the separator due to the methyl groups. Moreover, because of the lower polarity of methyl groups, as a battery separator, methyl cellulose can contact the electrolyte sufficiently, improving the wettability of the separator. The experimental results obtained by Liao et al. [83] showed that the cloud point temperature and the surface morphology of the battery separator moved to higher values and changed from dense to porous with increasing MC content, respectively. The hybrid battery separator also has higher electrical absorption than the pure HDPE battery separator. Xiao and coworkers [152] prepared lithium battery separators by coating polyvinylidene fluoride (PVDF) on the surface of a methyl cellulose (MC)-based membrane. The external PVDF coating is porous, which can effectively improve electrolyte absorption, and the lithium-ion migration number is much larger than that of a pure methyl cellulose battery separator.

#### 2.2.3. Carboxymethyl Cellulose

**Figure 7:**
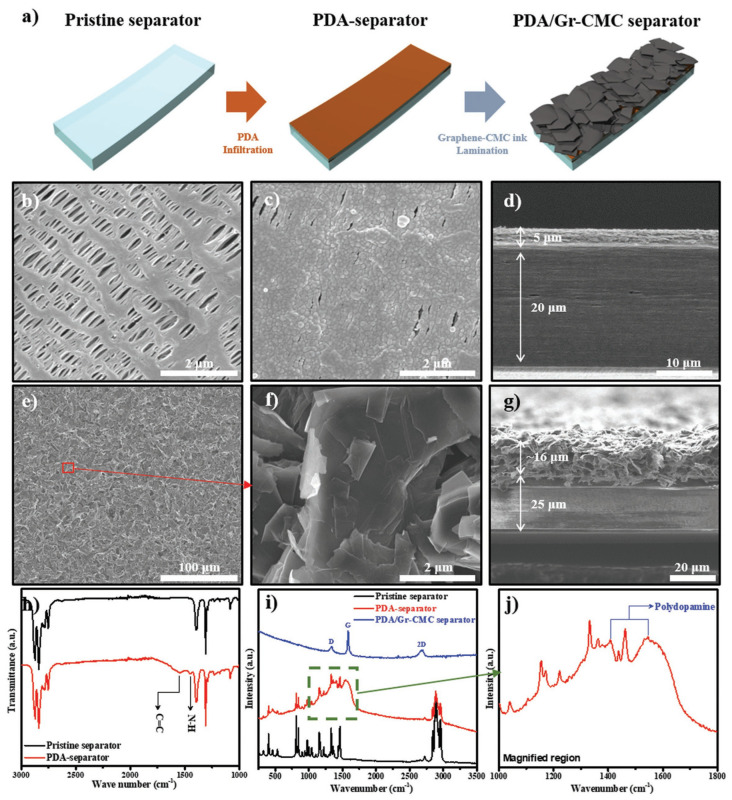
Specific working diagram and related data graph [157]. (**a**) Schematic to illustrate the preparation step of a PDA/Gr-CMC separator; SEM images of (**b**) a pristine separator and (**c**) a PDA-separator; (**d**) cross-section view of the PDA-separator; (**e**,**f**) SEM images of a PDA/Gr-CMC separator; (**g**) cross-section view of the PDA/Gr-CMC separator; (**h**) FT-IR spectra; (**i**,**j**) Raman spectra analysis. Copyright © 2018, with permission from WILEY-VCH Verlag GmbH & Co. KGaA, Weinheim.

Carboxymethyl cellulose (CMC) is one of the most promising cellulose derivatives, due to its unique surface properties, mechanical strength, adjustable hydrophilicity, adhesive properties, availability and richness of raw materials, and low-cost synthesis processes [153,154,155]. Carboxymethyl has excellent thermal stability and hydrophilicity, so carboxymethyl cellulose can improve the thermal stability and electrolyte absorption rate of the battery separator when used as a battery separator. Sun et al. fabricated a biopolymer hybrid electrolyte (BBE) separator by mixing commercial methyl cellulose (MC) and carboxymethyl cellulose (CMC) [84]. The obtained battery separator has excellent ionic conductivity (1.20 mS·cm^−1^), tensile strength (11.02 MPa), elongation at break (17.33%), and thermal decomposition temperature (202.84 °C). Shi and coworkers coated a layer of Al_2_O_3_ on the surface of the PE battery separator using water as a solvent and styrene butadiene rubber and carboxymethyl cellulose as binders. The introduction of a ceramic coating gave the battery separator excellent thermal stability, while the addition of CMC effectively improved the electrolyte wettability [156]. Kim and Pol [157] first reported a two-layer multifunctional battery separator with polydopamine (PDA) and graphene carboxymethyl cellulose (Gr-CMC) deposited on a standard polypropylene diaphragm that provides excellent electrolyte wetting, enhanced conductivity, and Li storage capacity, advantages that promote excellent and efficient electrochemical reactions in Li/LiFePO_4_ (LFP) full cells and kinetics. When the PDA/Gr-CMC diaphragm is used in a battery test system, it exhibits significantly improved cycling stability and coulombic efficiency, and significantly lower interfacial impedance between the electrode and separator (Figure 7). Kennedy et al. [85] produced a ceramic-coated composite separator (CCS) using alumina sodium carboxymethyl cellulose and polyvinyl alcohol to stabilize the coating slurry and optimize the slurry preparation process. CMC effectively controls the viscosity of slurry, maintains stable slurry dispersion, and enhances adhesion strength, thermal stability, and electrolyte wettability.

#### 2.2.4. Ethyl Cellulose

**Figure 8:**
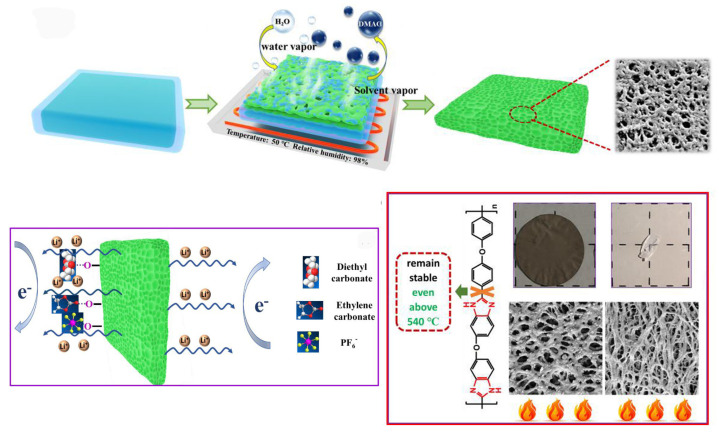
Schematic drawing of separators based on PBI and EC [163]. Copyright © 2019, with permission from American Chemical Society.

Ethyl cellulose (EC) is a multifunctional, water-insoluble cellulose ether with high thermal stability and mechanical properties. Ethyl cellulose has more polar groups, which makes it have good wettability in electrolytes [158,159,160,161,162]. Ethyl cellulose has better thermal and chemical resistance due to the introduction of ethyl groups, and the moderate polarity of ethyl groups can increase the electrolyte uptake of the separator. Therefore, lithium-ion battery separators using ethyl cellulose possess good thermal stability and electrolyte uptake. Xiong and colleagues applied a high-porosity ethyl cellulose coating on the polyolefin separator, and the results showed that the ethyl cellulose coating improved the thermal stability of the polyolefin separator and its wettability in the electrolyte [86]. The blended separator of poly (oxyphenylene benzimidazole) (PBI) and ethyl cellulose (EC) prepared by Chen et al. exhibited good heat resistance and good electrochemical properties [163]. The prepared co-blended polymer gel separator presented no significant dimensional change after 30 min at 350 °C, while the polyethylene (PE) battery separator almost completely melted. In addition, the ion conductivity of the PBI/EC separator is also much higher than that of the PE separator (Figure 8).

#### 2.2.5. Hydroxy Cellulose

The hydroxy cellulose commonly used in lithium-ion battery separators includes hydroxyethyl cellulose (HEC) and hydroxypropyl methyl cellulose (HPMC). For hydroxyethyl cellulose, the hydroxyethyl groups in its structure can be added to specific positions in the molecule by chemical decoration, thereby changing the properties and functions of the separator, such as improving the thermal stability and electrolyte uptake of the separator. For hydroxypropyl methyl cellulose, the groups in its structure have good wettability and play the role of transporting lithium ions, thus improving the ion conductivity and electrolyte absorption rate, and reducing the impedance of the separator itself.

**Figure 9:**
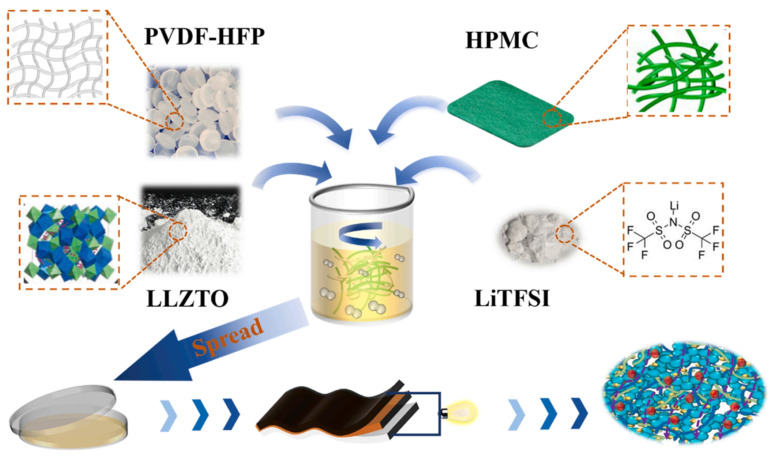
Mechanism and preparation diagram of HPMC battery separator [165]. Copyright © 2023, with permission from Elsevier.

Liao and colleagues used liquid paraffin as a porogen to prepare a hydroxyethyl cellulose aerogel coating-modified PP separator by the freeze-drying ethanol extraction method. The HEC aerogel coating after liquid paraffin extraction had a dense porous structure, which increased the specific surface area of the coating and effectively improved the liquid absorption rate of the PP separator [164]. Gao and coworkers [165] reported a novel lithium battery (PLHL-CSE) membrane consisting of polyvinylidene fluoride-co-hexafluoropropylene (PVDF-HFP), hydroxypropyl methyl cellulose (HPMC), lithium bis (trifluoromethanesulfonate) imide (LiTFSI), and Li_6.4_La_3_Zr_1.4_Ta_0.6_O_12_ (LLZTO), which showed better mechanical strength (3.0 MPa), enhanced ionic conductivity (0.25 mS/cm at 25 °C), and a larger lithium-ion mobility number (0.7). After 300 cycles at 1C, the cell showed high-capacity retention (95.6%) (Figure 9).

For chemically modified cellulose, using the introduction of surface groups on cellulose to improve the performance of lithium-ion battery separators is a common method at present. Generally speaking, increasing hydrophilic groups can improve the electrolyte uptake of the separator. Introducing stability and corrosion-resistant groups can increase the thermal stability and corrosion resistance of the battery separator, and attaching groups with transport functions can reduce the impedance of the separator itself and improve the ion conductivity. Currently, preparing battery separators with chemically modified cellulose has great development potential. Chemically modified cellulose makes the various properties and stability of the separator greatly improved, and their preparation price will not increase significantly compared with physically modified cellulose. Therefore, one development trend of cellulose lithium battery separators in the future is cellulose functionalization, to improve the various properties of the separator.

## 3. Preparation Method of Cellulose-Based Lithium Battery Separator

Cellulose-based battery separators can be prepared by two main types of methods: wet and dry. Wet methods include papermaking, coating, flow casting, vacuum filtration, and phase separation. Dry methods include aerogel and electrospinning. Both methods can produce high-quality cellulose-based battery separators with different structures and properties. However, dry methods have some advantages over wet methods, such as avoiding chemical reactions or organic solvents, and being more environmentally friendly and recyclable.

### 3.1. Wet Process Membrane Production

Wet methods are simple and low-cost techniques for preparing cellulose battery separators. They allow the adjustment of the separator thickness and porosity by controlling the solution or slurry concentration, coating thickness, baking time, and other parameters. This enables the production of customized separators with different sizes, shapes, and properties to match various battery applications. Moreover, wet methods can produce cellulose separators with smooth surfaces, which can reduce the impedance and improve the battery performance. Therefore, wet methods are feasible and advantageous for cellulose battery separator fabrication.

#### 3.1.1. Papermaking Method

**Figure 10:**
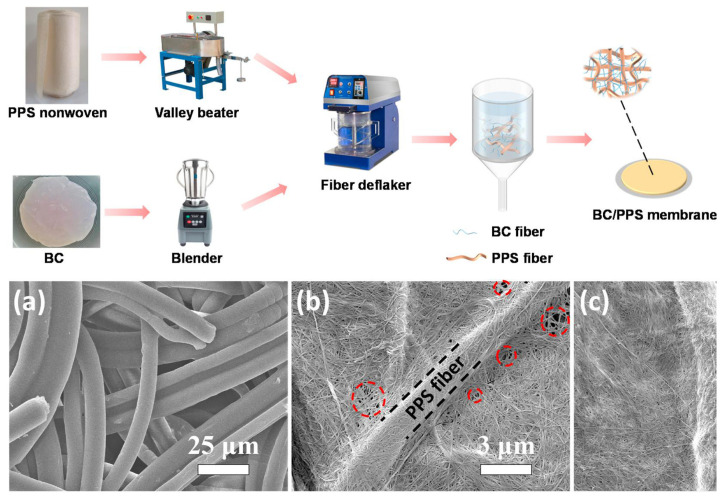
Preparation process of papermaking method [78]. SEM images of (**a**) PPS membrane, (**b**) 15%BC/PPS separator and (**c**) 20%BC/PPS separator. Due to the insufficient BC content, a few defects occurred in 15%BC/PPS separator (circled in red, Figure 10b). Copyright © 2021, with permission from Elsevier.

The papermaking method, also known as the wet non-woven method, is a technique that follows the basic principle of paper formation. It involves filtering and forming the flocs in the liquid phase. This method has the advantages of mature technology, high production efficiency, safety, and low cost, making it suitable for large-scale production [166]. Polyphenylene sulfide (PPS) has excellent thermal stability, inherent flame retardancy, excellent chemical resistance, and low toxicity. However, how to prepare polyphenylene sulfide battery separators with uniform microporous structures is still a problem. Zhu et al. [78] regulated the pore size of the composite separator by introducing BC nanofibers and improved its ability to form a separator. The BC/PPS composite separator exhibited remarkable thermal stability, good electrolyte wettability, and outstanding ionic conductivity. It has excellent rate performance and cycling stability (Figure 10). Zhang and coworkers first beat the natural green biomass material to remove impurities, and then added silicon and flame retardant into the pulp. They then prepared a non-woven membrane through a papermaking method, effectively improving the heat resistance and flame retardancy of the natural cellulose separator [167]. This separator exhibits better liquid absorption, interface stability, and ion conductivity. Similarly, Huang et al. developed a universal and cost-effective strategy to manufacture a highly thermally stable separator by coating natural halloysite nanotubes (HNTs) on both sides of commercial paper or waste newspapers [168]. The obtained separator presented excellent performance, including thermal stability, thermal conductivity, wettability, and electrolyte absorption. Compared with commercially available PP separators, even after being treated at 180 °C, the composite separator still exhibited a normal structure, and the assembled battery could operate normally. In addition, the spike test of the soft pack battery indicated that this separator can prevent the thermal runaway of LIBs. Pan and colleagues introduced a novel three-layer diaphragm design that significantly improved the cycle stability and safety of lithium-based batteries [169]. They used a simple papermaking process to produce thin, thermally stable, soft, and hydrophilic cellulose nanofiber layers directly laminated on each side of the plasma-treated polyethylene (PE) separator. When the internal PE layer melts, even at 200 °C, the three-layer separator can maintain its dimensional stability and prevent ion transfer through the separator, thus providing an effective thermal shutdown function. Therefore, the current three-layer separator design based on nanocellulose greatly promotes the implementation of high-energy-density lithium-based batteries. However, the papermaking method is only applicable to non-water-soluble cellulose suspensions, and the process of papermaking has more factors affecting the performance of the separator. First of all, the degree of beating determines whether the thickness of the separator is uniform and whether the mechanical properties are excellent. The higher the beating degree, the finer the fiber fraction, the greater the specific surface area, the more uniform the dispersion of the suspension, the greater the bonding force between the prepared separator fibers, the better the mechanical properties, and the better the wettability of the electrolyte. But at the same time, the more closely the fibers are stacked, the pore size and porosity will decrease. Therefore, it is necessary to control the appropriate beating degree to obtain the maximum porosity and at the same time ensure the strength of the separator.

#### 3.1.2. Coating Method

**Figure 11:**
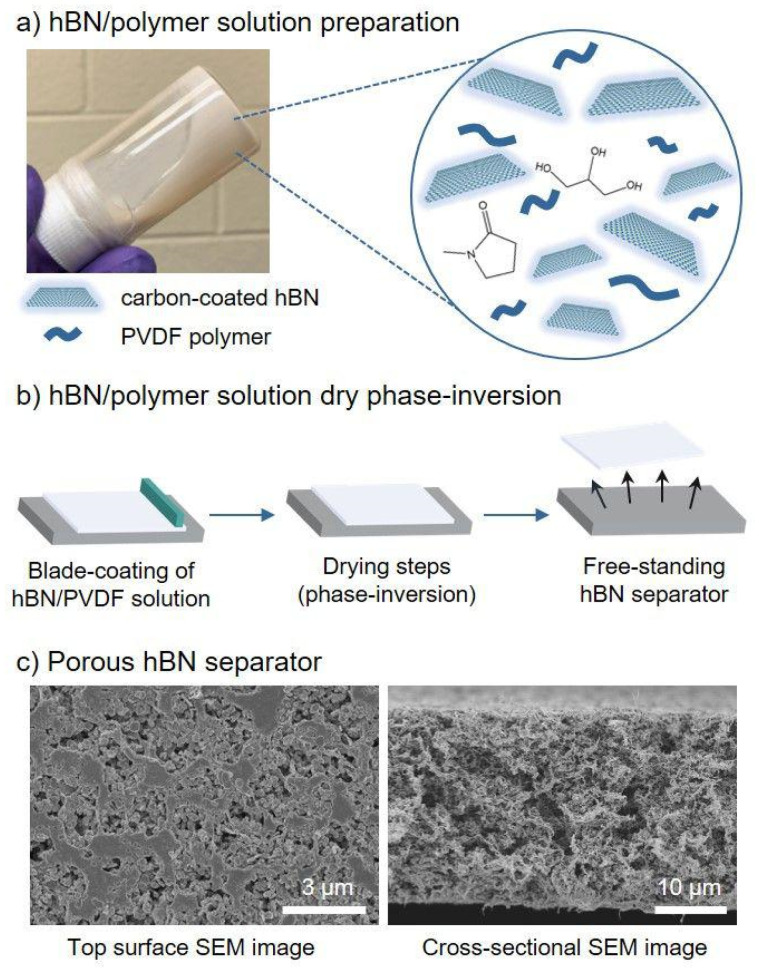
Schematic diagram of the effect of coating methods for preparing battery separators [178]. Copyright © 2020, with permission from American Chemical Society.

The coating method refers to coating organic or inorganic materials or organic–inorganic hybrid materials on the separator surface to improve the thermal stability of the separator and the wettability of the electrolyte, aiming to improve the performance of the lithium battery (Figure 11).

The commonly used inorganic coating materials include SiO_2_, Al_2_O_3_ and TiO_2_, etc. As these materials are thermally stable, when combined with the cellulose matrix, the resulting cellulose composite separator has both the flexibility of organic materials and the rigidity of inorganic materials. This can effectively inhibit the deformation of the separator at high temperatures [170]. Zhu and colleagues uniformly deposited thin titanium dioxide layers on the surface of a commercial polypropylene (PP) diaphragm by a simple sol–gel coating method that is easy to prepare and does not require an adhesive [171]. The thin TiO_2_ layer considerably improves the thermal stability of the separator, and there is no significant shrinkage when the separator is heated at 170 °C for 30 min. Also, the wettability of the modified electrolyte is greatly improved, which increases the Li^+^ migration rate. The LiFePO_4_/Li cell with a PP/TiO_2_ separator shows excellent multiplicative performance and cycling performance at high currents. In particular, it has a high capacity of 92.6 mAh·g^−1^ at 15 C. The improved separator enhances both the safety and electrochemical performance of the battery, which will help to achieve the preparation of high-safety, high-capacity Li-ion batteries. Chen and coworkers [37] used a cellulose diacetate (CDA)-SiO_2_ composite coating to prepare lithium battery separators. The electrolyte absorption rate and thermal stability of the diaphragm were improved. The pore structure of the composite coating can be adjusted by the weight ratio of the SiO_2_ precursor tetraethoxysilane (TEOS) in the coating solution. The composite separator exhibited the best overall performance when the weight ratio of TEOS in the coating solution was 9.4%. Xu and colleagues [172] successfully prepared BC/Al_2_O_3_ composite diaphragms by coating Al_2_O_3_ on BC nanofibers, and tests showed that the diaphragms were thermally stable and deformation-free at 200 °C. In contrast, the commercial PP-PE-PP battery separator has a large shrinkage rate. In addition, the separator has large porosity and excellent electrolyte uptake, resulting in greater ionic conductivity, lower interfacial resistance, and better electrochemical stability.

Common organic coatings include polyvinylidene fluoride, polydopamine, polyimide, polyborate, and styrene butadiene rubber. For example, Cao and colleagues introduced three popular organic polymers as coatings and evaluated their performance in lithium batteries with redox mediators [173]. Wang and coworkers [174] prepared a novel surface-modified PE/PVDF separator by coating PVDF organic particles on a conventional microporous polyethylene (PE) separator. Compared with the conventional PE separator, the PE/PVDF separator possesses higher porosity (61.4%), better electrolyte wettability (contact angle with water of 3.28° ± 0.21°), and superior ionic conductivity (1.53 mS·cm^−1^).

Researchers have found that the overall performance of the battery separator can be greatly improved by coating it with inorganic/organic coatings. This is because on the one hand, inorganic particles can be used to improve the flame retardancy and electrode interface compatibility of cellulose separators, and on the other hand, organic coatings can be used to improve the electrolyte wettability of cellulose separators [175,176]. Su and coworkers [177] prepared battery separators on a polyethylene (PE) substrate with SnO_2_ and hydroxypropyl methyl cellulose (HPMC), which had an initial discharge capacity of 142.3 mAh g^−1^ and a capacity retention of 77.9% after 250 cycles at 1C, which was higher than that of PE (140.6 mAh g^−1^ and 59.5% retention). The film can be formed on the Li foil in direct contact with the SnO_2_ layer, which reduces the Li nucleation overpotential and promotes eventual uniform Li deposition.

Coating methods mainly include the scraping coating method and immersion coating method. The advantages of the scraping coating method are simple operation and good product accuracy. However, the scraping method can lead to weak adhesion between the coating and the cellulose separator, which affects the mechanical strength of the separator. The advantage of the impregnation coating method is that the coating solution will penetrate the interior of the cellulose separator, with stronger binding force, but the thickness is difficult to control, and the coating solution will block the pores of the separator, thereby changing the porous structure and affecting the film-forming effect.

#### 3.1.3. Casting Method

**Figure 12:**
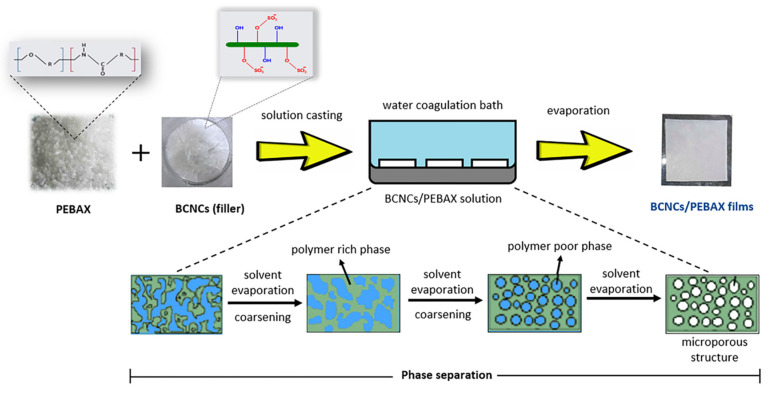
Schematic diagram of the formation of BCNC/PEBAX microporous membrane by flow casting method [77]. Copyright © 2023, with permission from Elsevier.

The principle of the casting method is to mix the required experimental raw materials uniformly and pour them into a glass container such as a surface dish, and then heat the solvent to evaporate or dry naturally. Separators prepared by the casting method have the advantages of uniform thickness, low cost, fewer impurities, and higher crystallinity. Compared to solid-state separators, gel polymer electrolytes have excellent mechanical stability, flexibility, enhanced ionic conductivity, and good interfacial contact with electrodes, providing an ideal alternative to conventional lithium battery separators [179]. Ajkidkarn and colleagues [77] used a thin-film casting method to prepare BCNC/PEBAX microporous films. The BCNC/PEBAX battery separator has excellent ionic conductivity and a porosity of 56.8% (Figure 12). Bolloli and coworkers [121] used nanocomposite materials from poly (vinylidene fluoride) (PVDF) as a matrix polymer and a stable DMF suspension of nanocrystalline cellulose (NCC) as the reinforcing phase to form porous and dense nanocomposite membranes by film casting methods, respectively.

The advantage of the casting method is that the film-forming process is simple, but its film-forming conditions are generally to be carried out in a heated environment, which could cause the full evaporation of organic solvents. On the other hand, cellulose is a highly polar polymer material. During the heating process, cellulose will accumulate tightly due to heating, making it difficult to form a loose porous structure. The porosity of the membrane is difficult to control, and it is often necessary to improve the porosity of the membrane by adding other modified materials to the cellulose solution or suspension.

#### 3.1.4. Vacuum Filtration Method

**Figure 13:**
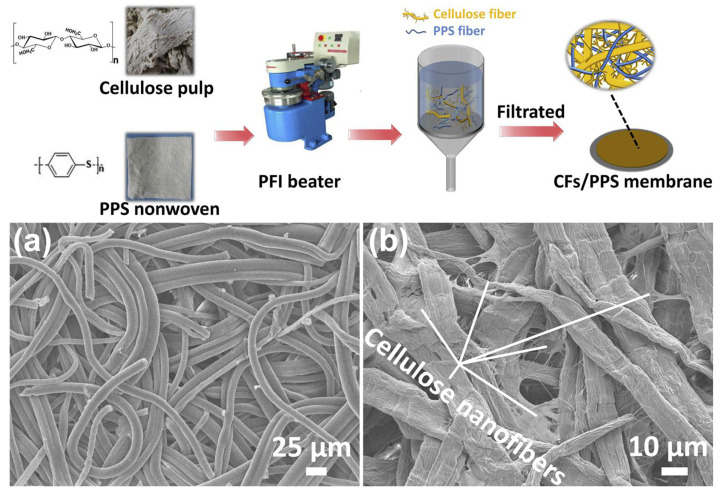
Schematic illustration for preparation of CF/PPS composite membrane by vacuum filtration method [182]. Typical SEM images of (**a**) PPS membrane, (**b**) cellulose membrane Copyright © 2021, with permission from Elsevier.

Compared to other preparation methods, the vacuum filtration method significantly improves the efficiency of preparing lithium battery separators, but the vacuum filtration method is only applicable to non-water-soluble cellulose. Lv et al. prepared a pure cellulose paper (CCP) lithium battery separator reinforced with cellulose nanofibers (CNFs) by the vacuum filtration method [180]. Lithium batteries assembled with CCP separator exhibit good cycling performance (with a capacity retention rate of 91% after 100 cycles). Huang and coworkers [181] prepared a composite nanofiber membrane containing bacterial cellulose (BC) and halloysite nanotubes (HNTs) using the vacuum filtration method. The bacterial cellulose film doped with HNTs exhibits excellent thermal stability, mechanical strength, and a liquid absorption rate of up to 369%. Zhu and his colleagues [182] introduced cellulose fibers (CFs) as a reinforcing material in order to improve the performance of PPS separator membranes. The large number of nanofibers in CFs helps to regulate the pore size of the membrane and enhance the mechanical properties of the membrane. In addition, the hydroxyl groups contained in CFs can be formed with the liquid electrolyte through hydrogen bonding, thus improving the wettability of the separator (Figure 13).

Vacuum filtration and papermaking share a similar membrane formation mechanism, but filtration offers higher efficiency and easier control over the membrane thickness. However, the hydrogen bonding between nanocellulose fibers leads to a dense packing of the fibers after filtration with water as the dispersion medium, resulting in a low porosity of the membrane. To improve the porosity, hydrophobic dispersion media can be added or the concentration of cellulose suspension can be reduced to create a looser arrangement of the fibers.

#### 3.1.5. Phase Separation Method

The phase separation method is generally divided into the thermally induced phase separation method [183,184,185] and non-solvent-induced phase separation method [186,187].

The thermally induced phase separation method is an important method for preparing porous polymer materials with specific structures [188,189,190,191]. Wu and colleagues [184] prepared a series of PVDF/PAN to blend separators via the thermally induced phase separation (TIPS) method for lithium-ion batteries. Li et al. [183] effectively manufactured a polypropylene (PP)/polyethylene (PE) multilayer separator with honeycomb-shaped submicron pore structures for lithium-ion batteries by combining multilayer co-extrusion (MC) and thermally induced phase separation (TIPS). The prepared separator, named MC-TIPS PP/PE, not only exhibits an effective thermal shutdown function and a wider closing temperature window but also exhibits higher thermal stability of 232.5 °C compared to separators with PP and PE.

**Figure 14:**
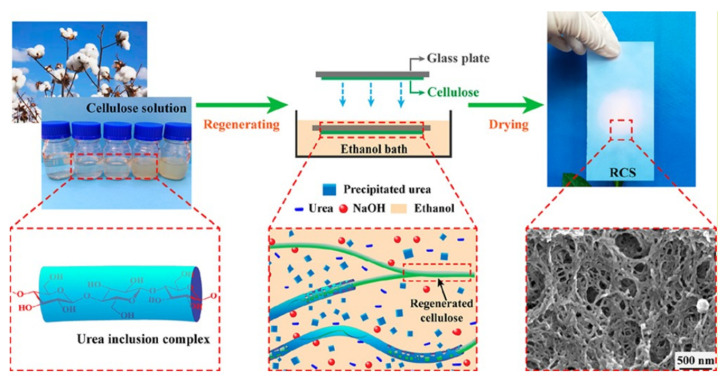
Preparation of highly porous battery separators using a phase separation method (NIPS) [196]. Copyright © 2021, with permission from American Chemical Society.

Non-solvent-induced phase separation (NIPS) is the process of dissolving polymers in solvents to form a homogeneous solution. At this time, reagents with stronger solubility with the solvent (called extractants) are slowly added to extract the solvent, forming a two-phase structure with the polymer as the continuous phase and the solvent as the dispersed phase. The solvent is then removed to obtain a microporous polymer [192,193,194,195]. Wang and colleagues [196] prepared a highly porous polybenzimidazole-based separator by using the NIPS method (Figure 14). The separator has a porosity of 92%, electrolyte absorption of 594 wt%, and a strong mechanical strength of 15.9 MPa. In addition, experimental tests (electrochemical analysis and XPS test) and density functional theory calculations indicate that the electron-rich imidazole ring of PPI can enhance the electrostatic attraction interaction of Li^+^ mobility and prevent the migration of PF^6-^ through electrostatic repulsion interactions. Zhang and coworkers [197] successfully obtained an ideal PI separator with high porosity and pore connectivity through the NIPS process, and used it for lithium-ion batteries (LIBs). In combination with extremely high thermal stability, the PI separator provides excellent wettability and high liquid electrolyte uptake in liquid electrolytes. With liquid electrolyte activation, the PI separator can achieve ionic conductivity up to 2.15 mS·cm^−1^. Cells assembled from PI separators possess lower resistance, higher discharge capacity, and better multiplier performance than cells with typical PP separators.

The phase separation method enables the control of porosity and pore size of lithium battery separators, producing highly coherent porous membrane materials. However, the method involves high-temperature heating, which causes the evaporation of porogenic small molecules and organic solvents into the air, posing threats to human health and environmental quality.

### 3.2. Dry Membrane Production

The dry method for preparing cellulose battery separators does not require organic solvents, which is beneficial for avoiding environmental issues and solvent residues.

#### 3.2.1. Aerogel Method

**Figure 15:**
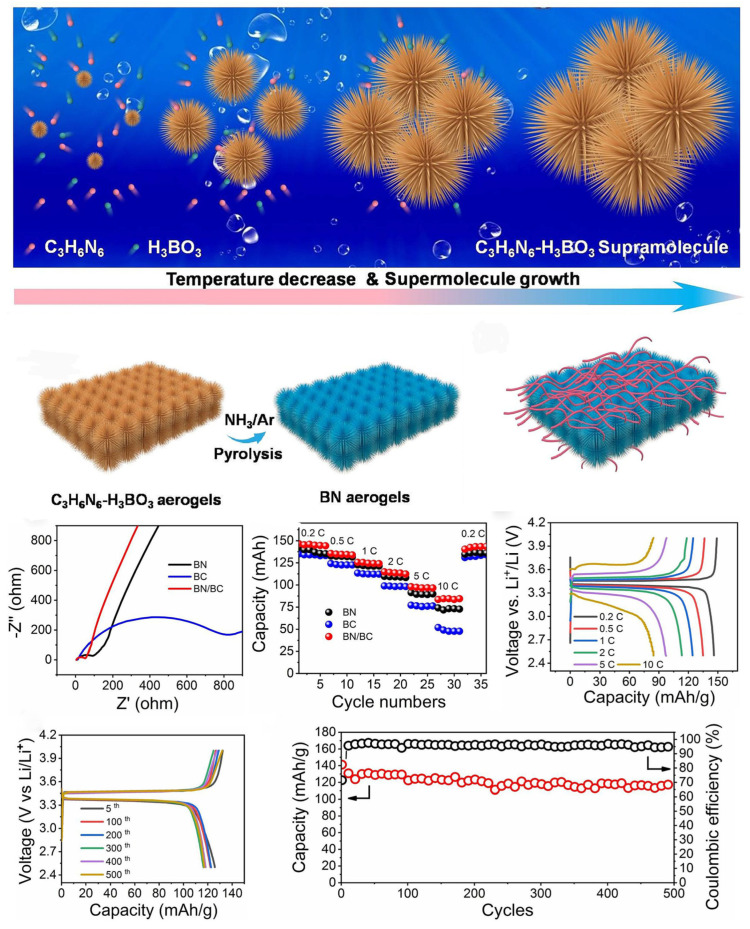
Aerogel cellulose-based battery separator [201]. Copyright 2023, with permission from Elsevier.

The aerogel method is used to convert a cellulose slurry into cellulose aerogel by the ultrasonic or spray method and then compress it into a membrane to form a cellulose separator [198,199]. To address the toxic solvents generated during the preparation of cellulose-based lithium battery separators, Liao et al. [164] prepared a cellulose aerogel-coated PP separator based on hydroxyethyl cellulose (HEC). Compared to their uncoated counterparts, porous cellulose aerogel-coated separators exhibit excellent dimensional stability and electrolyte uptake, resulting in higher ionic conductivity and better cycling performance. Raafat and colleagues [200] developed a highly flexible and environmentally friendly cellulose nanofiber aerogel (CNF-AG) separator and evaluated its dynamic behavior in a battery. The obtained separator had a mesoporous/macroporous ratio of 99.5%, as well as good mechanical stability, and its performance was superior to commercial glass fiber (GF) membranes. Boron nitride has excellent physical and chemical properties, and its coating on the surface of other materials can greatly improve thermal stability. Yang et al. [201] prepared a boron nitride (BN) aerogel by burning melamine–boric acid supramolecular hydrogel at a high temperature, and then compounded it with bacterial cellulose (BC) to form a BN/BC composite aerogel. The addition of BN makes the composite aerogel have excellent flame-retardant properties, and BC improves the mechanical strength of the composite aerogel and gives it more groups. The batteries assembled from them have excellent cycle stability and multiplication performance (Figure 15).

The aerogel method produces cellulose separators with high cellulose content, exceeding 95%, which enhances the stability. The separators have a nanoporous structure that provides ion channels, facilitating ion transport and improving the ion conductivity of the battery. The separators also have a uniform thickness that prevents the lithium dendrites from puncturing the lithium-ion battery.

#### 3.2.2. Electrospinning Method

**Figure 16:**
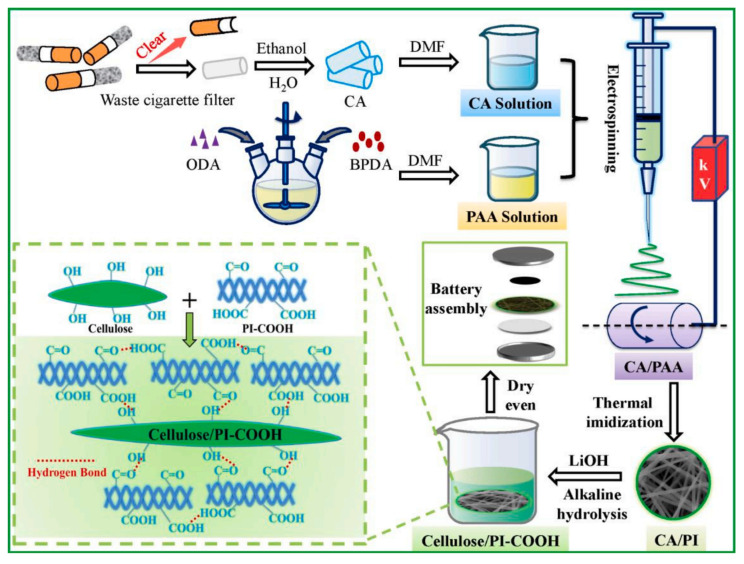
Lithium-ion battery separator prepared by electrospinning [210]. Copyright © 2021, with permission from Elsevier.

Electrospinning could produce fibers with a very fine fiber diameter and large specific surface area. The layered film formed by stacking these fibers exhibits excellent ion conductivity and high porosity. Therefore, electrospinning technology has a wide range of applications in the preparation of lithium battery separators [202,203,204,205,206,207,208]. Boriboon et al. [209] developed a cellulose acetate/titanium dioxide composite separator by the electrospinning method. The addition of titanium dioxide increased the thermal decomposition temperature of the cellulose membrane to a certain extent, the added titanium dioxide particles effectively improved the electrolyte wettability of the separator, and the lithium migration number increased from 0.22 to 0.62. The lithium-ion battery with the composite separator was able to maintain a discharge capacity of 79 mAh/g after 30 cycles. Deng and coworkers [210] prepared a novel environmentally friendly hydrogen-bonded (H-bonded) cross-linked cellulose/carboxylated PI (Cellulose/PI-COOH) nanofiber composite diaphragm by electrostatic spinning, and the three-dimensional interconnected structure generated by the H-bonded cross-linking was beneficial for improving the mechanical properties of the composite diaphragm. The original PI cell diaphragm (6.8 MPa) can provide a tensile strength of 34.2 MPa compared to the Cellulose/PI-COOH separator, and the ionic conductivity can reach 0.51 mS cm^−1^. The significantly enhanced tensile strength, flexibility, thermal stability, and flame retardancy of the Cellulose/PI-COOH separator greatly enhance the safety performance of the obtained LIB (Figure 16).

Electrospinning produces separators with a high specific surface area and a highly interconnected porous structure, unlike other film production methods. However, the separators have low crystallinity and low orientation between fibers, reducing the internal resistance of the separator but also decreasing the bonding points between fibers. This leads to poor mechanical properties that often fail to meet the assembly requirements of lithium batteries and need further optimization by modification methods such as hot pressing, mixing with highly polar organic substances, or combining with impregnation coating. Table 3 shows cellulose-based lithium battery separators prepared by different approaches.

**Table 3:** Cellulose-based lithium-ion battery separators prepared by different methods.

Preparation Method	Cellulose and Its Derivatives	Thickness (um)	Thermal Stability (°C)	Tensile Strength (MPa)	Ionic Conductivity (mS cm^−1^)	Porosity (%)	Cyclic Performance (%)	Electrolyte Absorption (%)	Ref.
Papermaking method	CMC	67	300	27.7	/	75	150 cycles 95.6	286	[166]
	BC/ATP	/	350	27.09	1.75	59.17	300 cycles 94.5	470	[211]
	CF/ANF	40	200	33	0.75	49.5	100 cycles 89.6	157	[212]
Coating method	PVDF/EC	/	347	73	0.79	/	200 cycles 95.7	/	[34]
	GF/CNF	30	/	/	1.7	66	200 cycles 80	/	[213]
Casting method	BCNC/ PEBAX	/	150	14.9	9.79	56.8	/	101.4	[77]
Vacuum filtration method	ECM	12	290	/	0.35	53	100 cycles 78.6	281	[214]
	BC/CS	57.4	260	9.9	2.9	65.8	100 cycles 90	358	[215]
	ZIF-8/BC	/	325	50.1	1.12	73.2	100 cycles 89.2	340.5	[216]
	CFs/PPS-1/1	/	200	20.52	1.26	61.1	100 cycles 90.3	259.6	[182]
Electrospinning	PVDF/CA	30	450	7.6	1.36	86.5	50 cycles 91.8	311	[217]
	PVDF/TPP/CA	/	170	8.5	4.4	90	100 cycles 86.9	301	[218]
	PVDF/HFP/CA	/	200	34.1	6.16	66	100 cycles 75.4	355	[219]
Phase separation method	PVA/CNF	25	275	18	1.1	60	50 cycles 93	230	[220]
	PVDF/CNF/CEC	50	170	14.3	1.26	60.2	50 cycles 98.96	370	[221]
	RCS	19.74	260	/	1.25	61	100 cycles 81	436	[196]
Aerogel method	CNF/AG	160	150	/	2.64	99.5	200 cycles 41	12,000	[200]
	ANFs	/	300	/	1.04	92.6	200 cycles 70	695	[222]
	BN/BC	100	300	0.2	/	/	500 cycles 94	/	[201]

## 4. Conclusions and Outlook

### 4.1. Conclusions

By summarizing and generalizing the various methods of preparing cellulose separators, it can be known that when the preparation method is not conducive to adjusting the pore size, the performance of the battery separator will be greatly reduced, mainly because lithium ions cannot pass through the battery separator smoothly. When the pore size on the separator is smaller than the particle size of lithium ions, it will limit the normal passage of lithium ions. On the contrary, when the pore size on the separator is much larger than the particle size of lithium ions, it will cause the self-discharge of lithium batteries, resulting in a significant reduction in battery life. As can be seen from Table 3, if the preparation method can adjust the pore density and pore size of the cellulose-based battery separator (such as electrospinning), it can uniformly regulate the distribution of pores on the separator surface and allow lithium ions to quickly pass through the separator pores, reducing battery impedance and improving ion conductivity. At this time, the battery performance exhibited by the battery separator is the most excellent. The particle size of lithium ions is about 0.15 nm, so it is best when the pores on the battery separator are about 0.2–0.3 nm [223,224]. By this point, lithium ions can shuttle freely between the positive and negative electrodes of the battery, reducing the impedance of the battery itself, and will not cause self-discharge of the battery due to excessive pore size, damaging battery life. When the pores on the separator are evenly distributed, the effect shown by the battery is best.

Cellulose, as a biodegradable and renewable environmentally friendly new material, has become a research hotspot in the field of lithium-ion battery separators due to its excellent thermal stability, liquid absorption, and affordable price. However, cellulose has defects such as low mechanical strength and chemical resistance, which also affect the development of lithium-ion battery separators to a certain extent. Therefore, the selection of excellent modified materials, improving the production process of cellulose lithium battery separators, preserving the excellent performance of cellulose separators based on limiting their shortcomings, and enhancing the overall performance of the separator have become necessary paths for the development of cellulose lithium-ion battery separators. Cellulose-based lithium-ion battery separators face many challenges for practical applications, which can be broadly categorized into improving the performance of lithium-ion batteries, applying high-performance cellulose-based separators to high-energy-density lithium batteries, replacing polyolefin separators with high-performance, low-cost cellulose-based separators, and developing industrial production methods for high-performance cellulose-based lithium-ion battery separators. The application of cellulose separators in lithium-ion batteries is attributed to their good thermal and electrochemical stability, and these properties help to improve the wettability of the electrolyte and the lithium-ion transport efficiency, thus enhancing the initial capacity and cycling performance of the batteries. Specifically, the cellulose separator’s high porosity and appropriate pore size distribution can effectively absorb and retain the electrolyte and improve ionic conductivity; at the same time, its good mechanical strength prevents short-circuiting during charging and discharging, increasing the safety and life of the battery. In addition, the chemical stability of the cellulose separator and its good compatibility with the electrolyte can reduce the side reactions, lower the internal resistance, and extend the battery life. However, the cycle life of lithium-ion batteries is not only affected by the lithium-ion transfer efficiency, but also by a combination of factors such as the structural stability of the electrode materials (to avoid material pulverization and structural damage), the decomposition of the electrolyte (to prevent by-product generation and SEI film overthickness), and the side reactions at the electrode/electrolyte interface (to reduce the consumption of the electrode materials and the deterioration of the electrolyte), as well as the defects and mechanical stresses in the manufacturing process. Therefore, these influencing factors must be considered comprehensively when optimizing the performance of lithium-ion batteries.

### 4.2. Outlook

For these challenges, we propose the following corresponding solution ideas:

(1) Improvement of the comprehensive performance of high-performance cellulose-based separators.

The comprehensive performance of battery separators was determined by a series of factors, such as pore size and pore distribution, porosity, wettability, electrolyte absorption, ionic conductivity, mechanical strength, thermal stability, electrochemical stability, etc. [225]. The manufacture of separators suitable for high-energy-density lithium batteries not only requires controlling and adjusting the thickness and porous structure of the high-performance cellulose-based separators but also ensuring the thermal stability of the prepared separators, while having the mechanical properties of high-performance cellulose. Further search for suitable preparation methods and composite or modified materials that could be combined with high-performance cellulose is therefore needed to ensure that the separator achieves the required performance. There would be three main methods suitable for enhancing the performance of cellulose-based lithium battery separators: 1. Cellulose-based material aerogelation, which could improve the electrolyte absorption rate and porosity of the separator, and most importantly, could significantly improve the ionic conductivity. However, the capacity retention of lithium battery separators prepared by the aerogel method is not good: most of them are less than 70% after 200 cycles, and some are even as low as 40%. The capacity retention of the battery is an important measure of whether a battery can be used on a large scale. Therefore, it is especially important to improve the performance of aerogel and add some modified materials adapted to aerogel battery separators to enhance the capacity retention of lithium-ion batteries. 2. Selecting suitable cellulose-based materials is crucial for the performance of the battery separator. The above comprehensive studies revealed that cellulose-based materials such as cellulose acetate (CA), cellulose nanocrystals (CNCs), and bacterial cellulose (BC) have good performance as battery separators due to their surface hydrophilic groups and their internal electron-rich groups. Future battery separator substrates should have hydrophilic surfaces and electron-rich internal groups. 3. Introducing other materials with good thermal stability and high porosity, such as metal–organic frameworks (MOFs), into cellulose battery separators can improve their performance. MOFs are porous crystal materials with highly ordered nanopores that can serve as ion conductors to modulate ion transport. In the framework topology of MOFs, the open metal sites in the pores can chelate with anions in the electrolyte, thereby liberating the migration ability of cations. Moreover, they have good thermal stability and proton conductivity, which can improve the thermal stability and ion conductivity of battery separators [226,227,228].

(2) Expanding the application of high-performance cellulose-based separators in high-energy-density lithium batteries.

With the increasing complexity of portable electronics and the growing demand for electric vehicles and renewable energy storage, batteries with higher charge storage capacity and energy density are also required. However, conventional LIBs cannot provide high-performance energy storage devices due to the low theoretical capacity of the active electrode materials. Therefore, there is a need to develop new and more advanced rechargeable batteries. Currently, LSBs and aqueous batteries with high energy are attracting widespread attention [229,230,231,232,233]. But in aqueous batteries, the strong hydrophilicity of cellulose may lead to over-expansion of the separator, which affects the stability of its physical structure and performance. Secondly, during battery operation, chemical corrosion and structural damage caused by hydrogen precipitation reaction will weaken the mechanical strength of the cellulose separator, which will make it more likely to be ruptured or damaged during battery cycling, thus increasing the risk of battery short circuits. The high-performance cellulose-based separators that have been studied and prepared so far are mainly used for LIBs. Therefore, research on high-performance cellulose-based separators needs to keep pace with the times. The challenge of applying high-performance cellulose-based separators in high-energy-density lithium batteries lies in the ability of the separators to withstand the self-discharge and breakdown caused by the high energy density. Hence, enhancing the mechanical properties of high-performance cellulose-based separators is an important direction for future development. One possible approach may be to use chemically modified cellulose and introduce chemical groups that can improve the mechanical strength of high-energy-density lithium batteries.

(3) Exploring the potential of low-cost, high-performance cellulose-based materials with large-scale production to replace polyolefin materials.

Currently, most lithium-ion battery separators are made of polypropylene (PP) and polyethylene (PE), mainly because they can be mass-produced and their raw materials are cheap and easy to obtain. However, with the advancement of research, some cellulose-based materials have been found to be able to be mass-produced and applied, such as cellulose acetate (CA). CA not only has superior performance but can also greatly improve the performance of lithium-ion battery separators when applied. Moreover, it is cheap and suitable for large-scale applications. Therefore, replacing polyolefin separators with high-performance cellulose-based materials is feasible and can greatly enhance the overall performance of the current separators.

The main methods currently used to prepare high-performance cellulose-based separators are electrospinning, filtration, phase separation, and papermaking. Although most of the separators prepared by these methods have significantly improved performance, electrospinning is time-consuming and expensive, which limits its production efficiency. Filtration is simple and easy, but not suitable for large-scale production. Phase separation has many experimental parameters and difficult reaction conditions. In contrast, papermaking is the most promising preparation technology because of its simplicity, eco-friendliness, low cost, and scalability. However, there is less research on papermaking methods, mainly because there are strict requirements on the raw materials. Therefore, more attention should be paid to the development and optimization of preparation techniques and devices for continuous mass production.

All in all, cellulose-based lithium battery separators are promising green and eco-friendly materials for energy storage applications. They exhibit high specific surface area, high mechanical strength, high ionic conductivity, high thermal stability, and high wettability. Moreover, they are low cost, renewable, and biodegradable. Therefore, cellulose-based lithium battery separators have great potential for future development.

## Figures and Tables

**Table 1:** Comparison of cellulose-based battery separator performance with PP and PE separators.

Property	Cellulose-Based Separator	Polypropylene (PP) Separator and Polyethylene (PE) Separator
Environmental	Renewable, biodegradable	Nondegradable, long-term environmental impact
Thermal stability	High, does not shrink or melt at high temperatures	Shrinks or melts at high temperatures, increasing thermal runaway risk
Electrolyte wettability	Good, strong hydrophilicity	Poor, strong hydrophobicity, requires additional treatment
Mechanical strength	High, effectively prevents short circuits	Lower, easily damaged, increasing short circuit risk
Electrochemical stability	Good compatibility with electrolyte and electrode materials, fewer side reactions	Poor compatibility, may have side reactions
Production cost	Potentially higher, requires improved processes for large-scale commercialization	Lower, already mass-produced
Commercial maturity	In research stage, further validation needed for commercial use	Mature, widely used in current lithium-ion batteries

**Table 2:** Performance and cost comparison of polyolefin battery separators and cellulose-based lithium battery separators.

Kind	Materials	Preparation Method	Thickness (μm)	Tensile Strength (Mpa)	Thermal Stability (°C)	Electrolyte Absorption (%)	Porosity (%)	Ionic Conductivity (mS⋅cm^−1^)	Cost (¥)	Cyclic Performance (%)	Ref.
Physically modified cellulose	MFC	Papermaking process	35	61	150	93	46	0.4	140	50 cycles 99.5	[70]
	SWP@PET@MFC	Papermaking process	51.3	/	200	348.6	/	0.77	269	200 cycles 80.2	[71]
	MCC/PLA/PBS	Phase separation method	30	3–6	135	138	/	2.06	525	/	[72]
	CNF/PET	Papermaking process	30	3	257	250	70	/	220	100 cycles 91.7	[73]
	TBA-FD	Vacuum filtration method	/	13	160	296	70.8	1.90	120	100 cycles 96.4	[74]
	HAP/CNF	Vacuum filtration method	28	9.94	250	162	76	0.81	160	100 cycles 67.1	[75]
	MCNC	Phase separation method	150	/	/	275	75.3	2.7	235	50 cycles 99.8	[76]
	BCNCs/PEBAX	Phase separation method	/	14.9	/	101.4	56.8	9.79	260	/	[77]
	BC/PBS	Suction filtration method	60	3	285	216	62.7	1.55	430	100 cycles 92	[78]
	BLA	Casting method	85	12.9	210	446	70	2.45	315	100 cycles 97	[79]
Chemically modified cellulose	CA/PAN/HAP	Electrospinning	46	11.18	270	281	61	3.02	330	50 cycles 99.48	[80]
	CPT20	UV-induced polymerization	15	/	230	190	/	4.17	430	100 cycles 98.2	[81]
	CA55	Phase separation	/	/	250	331	62	0.0307	240	/	[82]
	HDPE/MC	Coating method	/	3.5	130	130	68	1.01	250	50 cycles 96	[83]
	CMC	Microwave method	/	11.02	202	/	/	0.12	180	/	[84]
	Al_2_O_3_ CCSs	/	26	/	130	125.5	/	0.982	190	200 cycles 96.5	[85]
	PP/EC/PE/PP	Coating method	28	/	300	/	/	0.13	325	100 cycles 99	[86]
	PVDF/HEC/PVDF	Coating method	58	21.5	290	135.4	/	0.88	240	145 cycles 100	[87]
Polyolefin	PE	Coating method	24	102.24	135	186.7	65.64	0.206	62	450 cycles 72	[28]
	PP	Coating method	25	13.24	90	/	55	/	62	/	[88]
	PE	Coating method	12	82	140	123	42	0.12	50	200 cycles 78.4	[89]
	PP Celgard 2400	Coating method	25	/	170	91	/	/	62	100 cycles 75	[90]
	PE	Coating method	24.8	94.3	140	119	43.5	0.212	65	200 cycles 83.1	[91]
	PP	Coating method	25	127	140	88	44	0.108	62	60 cycles 66	[92]
Synthetics	PVDF/PET	Coating method	22	18	160	72	26.3	0.003	110	/	[93]
	PET	Coating method	22	36.1	255	89	/	1.34	120	300 cycles 43.5	[94]
	PVDF/HFP	Electrospinning	58	2.74	200	350	92	2.05	230	500 cycles 84.5	[95]
	PVDF/PU	Phase separation	27	7.83	140	201.8	66.3	1.34	240	60 cycles 74	[96]
	PVDF/EVA	Coating method	25	70	200	/	/	0.9	210	100 cycles 98.4	[97]
